# Ortho­rhom­bic polymorph of (6,7-dimeth­oxy-1,2,3,4-tetra­hydro­isoquinolin-1-yl)methanol

**DOI:** 10.1107/S1600536811019866

**Published:** 2011-06-18

**Authors:** Aouicha Elkhamlichi, Mohammed Lachkar, Brahim El Bali, Michal Dusek, Karla Fejfarova

**Affiliations:** a Laboratory of Engineering of Organometallic and Molecular Materials, "LIMOM" URAC 19, Department of Chemistry, Faculty of Sciences, PO Box 1796, 30000 Fès, Morocco; b Laboratory of Mineral Solid and Analytical Chemistry "LCSMA", Department of Chemistry, Faculty of Sciences, University Mohamed I, PO Box 717, 60000 Oujda, Morocco; c Institute of Physics ASCR, v.v.i., Na Slovance 2, 182 21 Praha 8, Czech Republic

## Abstract

The asymmetric unit of the title compound, C_12_H_17_NO_3_, contains two mol­ecules with different conformations. It is a polymorph of the monoclinic form [El Antri *et al.* (2004 ▶). *Mol­ecules*, **9**, 650–657]; the samples were crystallized at different temperatures from the same solvent. In both structures, mol­ecules are linked by O—H⋯N hydrogen bonds, forming chains. The conformations of the chains and their packing differ markedly in the two polymorphs.

## Related literature

For background to polymorphism in drugs, see: Brittan (1999 ▶); Bernstein (2002 ▶). For background to alkaloids and their pharmaceutical properties, see: Bently (1998 ▶); Herbert (1985 ▶). Kitamura *et al.* (1994 ▶); He *et al.* (2000 ▶); Gray *et al.* (1989 ▶). For natural-product isolation techniques, see: Dalton (1979 ▶). For the monoclinic polymorph, see: El Antri *et al.* (2004 ▶).
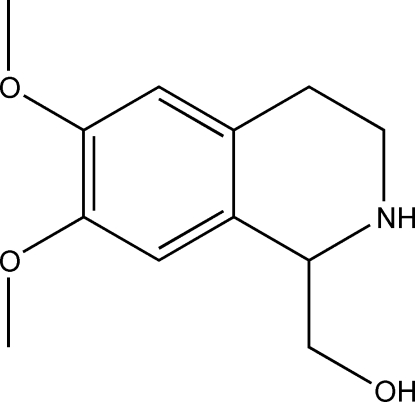


## Experimental

### 

#### Crystal data

C_12_H_17_NO_3_
*M* *_r_* = 223.27
Orthorhombic, 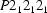

*a* = 8.9917 (11) Å
*b* = 13.4769 (12) Å
*c* = 18.576 (4) Å
*V* = 2251.0 (6) Å^3^
*Z* = 8
Mo *K*α radiation
μ = 0.09 mm^−1^
*T* = 150 K
0.51 × 0.35 × 0.32 mm

#### Data collection

Oxford Diffraction Xcalibur 2 diffractometer with a Sapphire 2 CCD detector
30187 measured reflections
2679 independent reflections
1515 reflections with *I* > 3σ(*I*)
*R*_int_ = 0.054

#### Refinement

*R*[*F* ^2^ > 2σ(*F* ^2^)] = 0.041
*wR*(*F* ^2^) = 0.091
*S* = 1.15
2679 reflections
301 parameters
H atoms treated by a mixture of independent and constrained refinement
Δρ_max_ = 0.24 e Å^−3^
Δρ_min_ = −0.15 e Å^−3^

### 

Data collection: *CrysAlis CCD* (Oxford Diffraction, 2005 ▶); cell refinement: *CrysAlis RED* (Oxford Diffraction, 2005 ▶); data reduction: *CrysAlis RED*; program(s) used to solve structure: *SIR2002* (Burla *et al.*, 2003 ▶); program(s) used to refine structure: *JANA2006* (Petříček *et al.*, 2006 ▶); molecular graphics: *DIAMOND* (Brandenburg & Putz, 2005 ▶) and *COOT* (Emsley *et al.*, 2010 ▶); software used to prepare material for publication: *JANA2006*.

## Supplementary Material

Crystal structure: contains datablock(s) global, I. DOI: 10.1107/S1600536811019866/hb5884sup1.cif

Structure factors: contains datablock(s) I. DOI: 10.1107/S1600536811019866/hb5884Isup2.hkl

Supplementary material file. DOI: 10.1107/S1600536811019866/hb5884Isup3.cml

Additional supplementary materials:  crystallographic information; 3D view; checkCIF report

## Figures and Tables

**Table 1:** Hydrogen-bond geometry (Å, °)

*D*—H⋯*A*	*D*—H	H⋯*A*	*D*⋯*A*	*D*—H⋯*A*
O1—H1*o*⋯N2^i^	0.83 (3)	1.92 (3)	2.740 (4)	169 (3)
O4—H4*o*⋯N1^ii^	0.79 (3)	2.03 (3)	2.810 (4)	172 (4)

Symmetry codes: (i) 
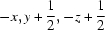
; (ii) 
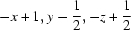
.

**Fig. 1.**
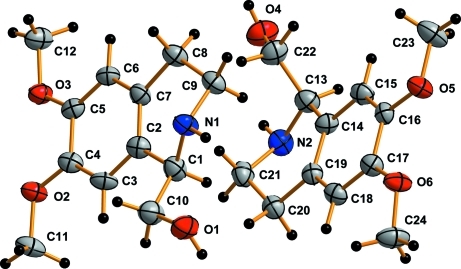
:. Ellipsoid plot of the title compound. Anisotropic displacement parameters drawn at the 50% probability level.

**Fig. 2.**
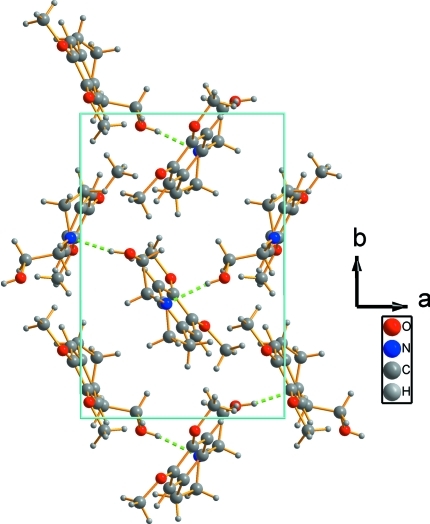
: Projection onto (001) of the structure of (I), H-bonds as dashed lines.

**Fig. 3.**
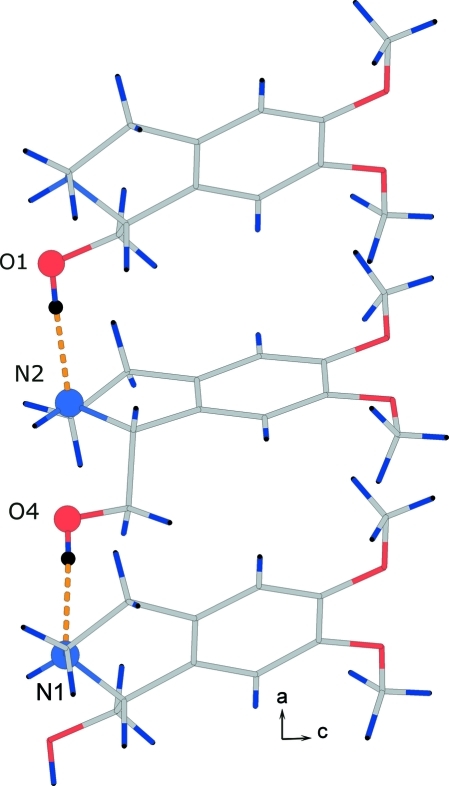
a&b: Orientation of the molecular chains in the monoclinic and orthorhombic forms. H-bonds as dashed lines.

**Fig. 4.**
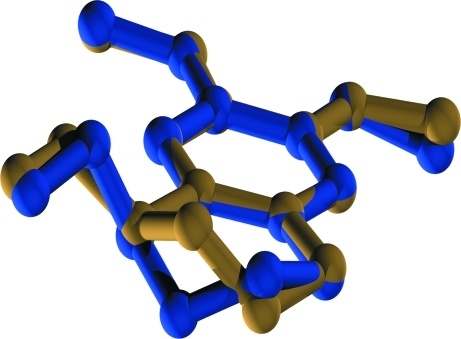
Overlay of two molecules present in assymetric unit. Yellow: C1–C12, blue: C13–C24. Hydrogen atoms are omitted for clarity.

## supplementary crystallographic information

### Comment

Temperature-induced polymorphism is important in pharmaceutical industry and especially, in the study of drug action with respect to their chirality (Brittan, 1999 and Bernstein, 2002). Isoquinoline alkaloids form a large group of compounds which could be extracted from many plants (Bently, 1998). Most of these natural alkaloids are optically active and allow interesting clinical uses such as analgesics, antihypertensives, smooth or skeletal muscle relaxants, antispasmodics, antitussives, antimalarials, narcotics and antipyretics (Kitamura *et al.*, 1994). Worthy also to report that 1-substituted tetrahydroisoquinolines display interesting biological and pharmacological properties (He *et al.*, 2000). Especially 1-methyl- and 1-phenyltetrahydroisoquinoline are involved in the treatment of Parkinson and other nervous system diseases (Gray *et al.*, 1989).

In a previous study (El Antri *et al.*, 2004), a single-crystal of 1-hydroxymethyl-7–8-dimethoxy-1,2,3,4-tetrahydroisoquinoline was measured at 173 K. A monoclinic symmetry (S. G.: *P*2_1_) has been found in the structure. Re-measurement, at 150 K, of another crystal from the same plant extract but crystallised at a different temperature, revealed the same composition C_12_H_17_NO_3_ (Fig. 1) but with an orthorhombic symmetry (S. G.: *P*2_1_2_1_2_1_). Testing measurements between 120 K and room temperature revealed however no phase transition. Moreover, no simple transformation between the monoclinic and orthorhombic unit cells could be found. Thus, 1-hydroxymethyl-7–8-dimethoxy-1,2,3,4-tetrahydroisoquinoline exists in two crystallographic forms. We report in the present study on the crystal determination of the new orthorhombic 1-hydroxymethyl-7–8-dimethoxy-1,2,3,4-tetrahydroisoquinoline. As in the monoclinic form, the molecules C_12_H_17_NO_3_ are connected by strong hydrogen bond O—H···N (Fig. 2). In the monoclinic previously published structure the chain runs along a and the molecules have alternating orientation with respect to the projection of hydrogen bonds into the *a* axis (Fig. 3a). On the other hand, in the orthorhombic structure reported here the chain direction is along b and the molecules are oriented in one side with respect to the projection of hydrogen bonds into the *b* axis (Fig. 3 b). The packing of the chains is quite different in both structures. H-bonds interactions are shown in Figs 3a&b as dashed lines and, for the new structure, they are summarized in Tab.3.

The asymmetric unit of the title compound contains two independent molecules, which differ in conformation of the tetrahydroisoquinoline ring (Fig. 4). The nitrogen atoms in each of the molecules are oriented on opposite sides of the ring. The conformation of the molecules may be influenced by intermolecular O—H···N hydrogen bonding involving both N1 and N2 atoms.

Distances and angles are of the same magnitude in the two crystals. The heterocyclic ring of tetrahydroisoquinoline adopts a half chair conformation. The conformation of the asymmetric carbon atoms are the same in both asymmetric molecules.

### Experimental

Alkaloids under investigations were extracted from the seeds of Calycotome villosa (Poiret) Link Subsp as in (El Antri *et al.*, 2004). The same procedure was used for extractions and crystals synthesis. However, the experiments took place at different room temperatures.

### Refinement

Positions of hydrogen atoms bounded to nitrogen and oxygen were refined using a distance restraint. The other H atoms were fixed in the ideal geometry. The methyl H atoms were allowed to rotate freely about the adjacent C—O bonds. The isotropic atomic displacement parameters of hydrogen atoms were evaluated as 1.2×*U*_eq_ of the parent atom.

### Crystal data

**Table:**

C_12_H_17_NO_3_	*F*(000) = 960
*M**_r_* = 223.27	*D*_x_ = 1.317 Mg m^−^^3^
Orthorhombic, *P*2_1_2_1_2_1_	Mo *K*α radiation, λ = 0.71069 Å
Hall symbol: P 2ac 2ab	Cell parameters from 9027 reflections
*a* = 8.9917 (11) Å	θ = 2.5–26.5°
*b* = 13.4769 (12) Å	µ = 0.09 mm^−^^1^
*c* = 18.576 (4) Å	*T* = 150 K
*V* = 2251.0 (6) Å^3^	Irregular, colorless
*Z* = 8	0.51 × 0.35 × 0.32 mm

### Data collection

**Table:**

Oxford Diffraction Xcalibur 2 diffractometer with a Sapphire 2 CCD detector	1515 reflections with *I* > 3σ(*I*)
Radiation source: X-ray tube	*R*_int_ = 0.054
graphite	θ_max_ = 26.6°, θ_min_ = 2.5°
Detector resolution: 8.3438 pixels mm^-1^	*h* = −11→11
Rotation method data acquisition using ω scans	*k* = −16→16
30187 measured reflections	*l* = −23→23
2679 independent reflections	

### Refinement

**Table:**

Refinement on *F*^2^	124 constraints
*R*[*F*^2^ > 2σ(*F*^2^)] = 0.041	H atoms treated by a mixture of independent and constrained refinement
*wR*(*F*^2^) = 0.091	Weighting scheme based on measured s.u.'s *w* = 1/[σ^2^(*I*) + 0.0016*I*^2^]
*S* = 1.15	(Δ/σ)_max_ = 0.004
2679 reflections	Δρ_max_ = 0.24 e Å^−^^3^
301 parameters	Δρ_min_ = −0.15 e Å^−^^3^
0 restraints	

### Special details

**Table:**

Refinement. The refinement was carried out against all reflections. The conventional *R*-factor is always based on *F*. The goodness of fit as well as the weighted *R*-factor are based on *F* and *F*^2^ for refinement carried out on *F* and *F*^2^, respectively. The threshold expression is used only for calculating *R*-factors *etc*. and it is not relevant to the choice of reflections for refinement.The program used for refinement, Jana2006, uses the weighting scheme based on the experimental expectations, see _refine_ls_weighting_details, that does not force *S* to be one. Therefore the values of *S* are usually larger than the ones from the *SHELX* program.

### Fractional atomic coordinates and isotropic or equivalent isotropic displacement parameters (Å^2^)

**Table:**

	*x*	*y*	*z*	*U*_iso_*/*U*_eq_	
O1	0.2415 (2)	0.53891 (18)	0.36542 (11)	0.0430 (7)	
O2	0.4410 (2)	0.45708 (14)	0.02813 (11)	0.0351 (7)	
O3	0.6069 (2)	0.30148 (13)	0.02562 (11)	0.0333 (7)	
O4	0.2946 (2)	−0.04072 (18)	0.15204 (13)	0.0501 (8)	
O5	0.0403 (2)	0.05130 (14)	0.48188 (10)	0.0355 (7)	
O6	−0.0888 (2)	0.22154 (13)	0.47614 (11)	0.0369 (7)	
N1	0.4198 (3)	0.37470 (18)	0.34959 (13)	0.0347 (9)	
N2	0.0505 (3)	0.09105 (18)	0.15335 (14)	0.0377 (10)	
C1	0.3258 (4)	0.4076 (2)	0.28954 (16)	0.0375 (11)	
C2	0.4044 (3)	0.3779 (2)	0.21914 (17)	0.0319 (10)	
C3	0.3811 (3)	0.4319 (2)	0.15544 (16)	0.0335 (10)	
C4	0.4524 (3)	0.4070 (2)	0.09213 (16)	0.0298 (10)	
C5	0.5455 (3)	0.3232 (2)	0.09070 (15)	0.0281 (10)	
C6	0.5657 (3)	0.2694 (2)	0.15306 (16)	0.0291 (10)	
C7	0.4979 (3)	0.2972 (2)	0.21772 (17)	0.0301 (10)	
C8	0.5328 (3)	0.2388 (2)	0.28580 (16)	0.0344 (11)	
C9	0.4336 (3)	0.2665 (2)	0.34811 (16)	0.0349 (11)	
C10	0.3029 (4)	0.5168 (2)	0.29733 (16)	0.0437 (12)	
C11	0.3469 (3)	0.5421 (2)	0.02716 (16)	0.0416 (11)	
C12	0.7128 (4)	0.2228 (2)	0.02437 (17)	0.0428 (12)	
C13	0.1076 (3)	0.0400 (2)	0.21832 (16)	0.0370 (10)	
C14	0.0628 (3)	0.09610 (19)	0.28544 (16)	0.0300 (10)	
C15	0.0789 (3)	0.0489 (2)	0.35197 (16)	0.0335 (10)	
C16	0.0308 (3)	0.0923 (2)	0.41458 (15)	0.0272 (10)	
C17	−0.0383 (3)	0.1861 (2)	0.41197 (16)	0.0300 (10)	
C18	−0.0500 (3)	0.2338 (2)	0.34686 (16)	0.0310 (11)	
C19	0.0006 (3)	0.1904 (2)	0.28279 (16)	0.0288 (10)	
C20	−0.0159 (3)	0.2449 (2)	0.21245 (16)	0.0355 (11)	
C21	0.0785 (3)	0.1987 (2)	0.15450 (16)	0.0390 (12)	
C22	0.2709 (3)	0.0187 (2)	0.21388 (17)	0.0437 (11)	
C23	0.1144 (4)	−0.0419 (2)	0.48738 (16)	0.0497 (12)	
C24	−0.1827 (4)	0.3079 (2)	0.47324 (17)	0.0472 (12)	
H1	0.229284	0.377124	0.289174	0.045*	
H3	0.314106	0.487379	0.155936	0.0402*	
H6	0.627874	0.211478	0.15206	0.0349*	
H8a	0.523519	0.169103	0.276183	0.0413*	
H8b	0.634849	0.249268	0.298996	0.0413*	
H9a	0.337109	0.237386	0.341366	0.0419*	
H9b	0.477612	0.244005	0.392266	0.0419*	
H10a	0.396496	0.550278	0.292131	0.0524*	
H10b	0.237013	0.539716	0.26024	0.0524*	
H11a	0.343252	0.568724	−0.020729	0.0499*	
H11b	0.248601	0.523434	0.042067	0.0499*	
H11c	0.38554	0.591309	0.059487	0.0499*	
H12a	0.752635	0.216224	−0.023333	0.0513*	
H12b	0.791882	0.237308	0.057458	0.0513*	
H12c	0.665196	0.161952	0.038152	0.0513*	
H13	0.061923	−0.024214	0.221065	0.0444*	
H15	0.124753	−0.015423	0.353911	0.0402*	
H18	−0.093988	0.298619	0.345199	0.0372*	
H20a	0.012609	0.31302	0.218823	0.0425*	
H20b	−0.118321	0.244051	0.197835	0.0425*	
H21a	0.052486	0.226707	0.10869	0.0468*	
H21b	0.181588	0.210629	0.164728	0.0468*	
H22a	0.324653	0.07989	0.208995	0.0524*	
H22b	0.301603	−0.017143	0.256005	0.0524*	
H23a	0.120147	−0.061226	0.537046	0.0597*	
H23b	0.059956	−0.091076	0.46086	0.0597*	
H23c	0.212938	−0.036144	0.467955	0.0597*	
H24a	−0.214712	0.324831	0.520983	0.0566*	
H24b	−0.127992	0.36243	0.453042	0.0566*	
H24c	−0.267876	0.294193	0.443745	0.0566*	
H1n	0.374 (3)	0.391 (2)	0.3865 (16)	0.0416*	
H2n	0.100 (3)	0.065 (2)	0.1185 (16)	0.0452*	
H1o	0.150 (3)	0.547 (3)	0.3609 (18)	0.0516*	
H4o	0.378 (4)	−0.059 (3)	0.153 (2)	0.0601*	

### Atomic displacement parameters (Å^2^)

**Table:**

	*U*^11^	*U*^22^	*U*^33^	*U*^12^	*U*^13^	*U*^23^
O1	0.0483 (13)	0.0424 (12)	0.0382 (13)	0.0103 (13)	0.0071 (12)	−0.0063 (11)
O2	0.0428 (12)	0.0309 (11)	0.0314 (12)	0.0109 (11)	−0.0027 (11)	0.0004 (10)
O3	0.0382 (12)	0.0343 (11)	0.0275 (12)	0.0090 (10)	0.0026 (11)	−0.0006 (10)
O4	0.0534 (16)	0.0548 (14)	0.0420 (13)	0.0117 (14)	0.0041 (13)	−0.0150 (13)
O5	0.0462 (13)	0.0283 (11)	0.0320 (12)	0.0061 (10)	−0.0031 (11)	0.0022 (10)
O6	0.0453 (13)	0.0380 (12)	0.0275 (12)	0.0155 (11)	−0.0011 (12)	−0.0016 (10)
N1	0.0477 (18)	0.0299 (14)	0.0266 (16)	0.0080 (14)	0.0035 (15)	−0.0023 (12)
N2	0.0456 (18)	0.0375 (16)	0.0300 (17)	−0.0004 (14)	0.0011 (14)	−0.0043 (13)
C1	0.046 (2)	0.0340 (18)	0.0328 (18)	0.0017 (16)	0.0025 (18)	0.0002 (15)
C2	0.0337 (18)	0.0305 (16)	0.0316 (18)	0.0013 (15)	−0.0035 (17)	−0.0027 (14)
C3	0.0390 (18)	0.0272 (17)	0.0345 (19)	0.0111 (14)	−0.0050 (16)	−0.0048 (15)
C4	0.0337 (17)	0.0296 (17)	0.0261 (18)	0.0010 (15)	−0.0089 (16)	−0.0028 (14)
C5	0.0261 (17)	0.0301 (17)	0.0281 (19)	0.0014 (15)	−0.0033 (16)	−0.0054 (14)
C6	0.0291 (18)	0.0274 (15)	0.0307 (19)	0.0043 (15)	−0.0019 (17)	−0.0025 (15)
C7	0.0340 (17)	0.0262 (16)	0.0301 (18)	0.0008 (14)	0.0000 (18)	−0.0002 (15)
C8	0.045 (2)	0.0289 (17)	0.0292 (18)	0.0029 (15)	−0.0014 (18)	−0.0017 (14)
C9	0.041 (2)	0.0311 (17)	0.032 (2)	0.0008 (16)	−0.0043 (18)	0.0029 (14)
C10	0.049 (2)	0.044 (2)	0.039 (2)	0.0104 (17)	0.0029 (17)	0.0029 (16)
C11	0.055 (2)	0.0323 (17)	0.0374 (19)	0.0135 (17)	−0.0089 (17)	0.0043 (17)
C12	0.050 (2)	0.049 (2)	0.0290 (17)	0.0168 (18)	0.0033 (18)	−0.0008 (16)
C13	0.0417 (19)	0.0336 (17)	0.0356 (19)	0.0008 (16)	0.0005 (17)	−0.0030 (17)
C14	0.0317 (17)	0.0310 (16)	0.0272 (18)	0.0030 (15)	0.0000 (16)	−0.0032 (14)
C15	0.0362 (18)	0.0256 (16)	0.0387 (19)	−0.0008 (16)	−0.0033 (16)	−0.0024 (15)
C16	0.0289 (17)	0.0273 (16)	0.0253 (18)	−0.0026 (14)	−0.0006 (15)	0.0013 (13)
C17	0.0336 (18)	0.0280 (17)	0.0283 (19)	−0.0001 (15)	−0.0018 (16)	−0.0026 (14)
C18	0.0345 (19)	0.0255 (16)	0.033 (2)	−0.0016 (15)	−0.0054 (17)	−0.0031 (15)
C19	0.0284 (17)	0.0314 (17)	0.0266 (17)	−0.0034 (14)	−0.0029 (17)	0.0013 (15)
C20	0.038 (2)	0.0336 (17)	0.034 (2)	−0.0040 (15)	−0.0028 (18)	0.0034 (15)
C21	0.039 (2)	0.046 (2)	0.032 (2)	−0.0051 (18)	−0.0013 (18)	0.0079 (15)
C22	0.046 (2)	0.047 (2)	0.0387 (18)	0.0051 (17)	0.0048 (18)	−0.0078 (18)
C23	0.076 (3)	0.0306 (17)	0.042 (2)	0.0087 (19)	−0.005 (2)	0.0031 (16)
C24	0.054 (2)	0.050 (2)	0.0378 (19)	0.0244 (19)	−0.001 (2)	−0.0094 (17)

### Geometric parameters (Å, °)

**Table:**

O1—C10	1.412 (4)	C9—H9b	0.96
O1—H1o	0.83 (3)	C10—H10a	0.96
O2—C4	1.371 (4)	C10—H10b	0.96
O2—C11	1.424 (4)	C11—H11a	0.96
O3—C5	1.361 (4)	C11—H11b	0.96
O3—C12	1.425 (4)	C11—H11c	0.96
O4—C22	1.416 (4)	C12—H12a	0.96
O4—H4o	0.79 (3)	C12—H12b	0.96
O5—C16	1.369 (3)	C12—H12c	0.96
O5—C23	1.425 (4)	C13—C14	1.513 (4)
O6—C17	1.362 (4)	C13—C22	1.498 (4)
O6—C24	1.439 (4)	C13—H13	0.96
N1—C1	1.468 (4)	C14—C15	1.398 (4)
N1—C9	1.464 (4)	C14—C19	1.389 (4)
N1—H1n	0.83 (3)	C15—C16	1.372 (4)
N2—C13	1.481 (4)	C15—H15	0.96
N2—C21	1.472 (4)	C16—C17	1.408 (4)
N2—H2n	0.86 (3)	C17—C18	1.374 (4)
C1—C2	1.539 (4)	C18—C19	1.402 (4)
C1—C10	1.493 (4)	C18—H18	0.96
C1—H1	0.96	C19—C20	1.507 (4)
C2—C3	1.405 (4)	C20—C21	1.506 (4)
C2—C7	1.375 (4)	C20—H20a	0.96
C3—C4	1.381 (4)	C20—H20b	0.96
C3—H3	0.96	C21—H21a	0.96
C4—C5	1.406 (4)	C21—H21b	0.96
C5—C6	1.379 (4)	C22—H22a	0.96
C6—C7	1.398 (4)	C22—H22b	0.96
C6—H6	0.96	C23—H23a	0.96
C7—C8	1.522 (4)	C23—H23b	0.96
C8—C9	1.508 (4)	C23—H23c	0.96
C8—H8a	0.96	C24—H24a	0.96
C8—H8b	0.96	C24—H24b	0.96
C9—H9a	0.96	C24—H24c	0.96
			
C10—O1—H1o	109 (2)	O3—C12—H12a	109.4718
C4—O2—C11	116.8 (2)	O3—C12—H12b	109.4712
C5—O3—C12	116.5 (2)	O3—C12—H12c	109.4707
C22—O4—H4o	108 (3)	H12a—C12—H12b	109.4716
C16—O5—C23	116.8 (2)	H12a—C12—H12c	109.4715
C17—O6—C24	116.5 (2)	H12b—C12—H12c	109.4704
C1—N1—C9	109.6 (2)	N2—C13—C14	110.3 (2)
C1—N1—H1n	105 (2)	N2—C13—C22	112.6 (2)
C9—N1—H1n	108.6 (19)	N2—C13—H13	108.2749
C13—N2—C21	112.7 (2)	C14—C13—C22	113.7 (2)
C13—N2—H2n	104 (2)	C14—C13—H13	107.0055
C21—N2—H2n	109 (2)	C22—C13—H13	104.4393
N1—C1—C2	107.6 (2)	C13—C14—C15	118.3 (2)
N1—C1—C10	107.7 (2)	C13—C14—C19	122.3 (3)
N1—C1—H1	113.3918	C15—C14—C19	119.3 (3)
C2—C1—C10	113.7 (2)	C14—C15—C16	121.5 (3)
C2—C1—H1	107.3416	C14—C15—H15	119.2572
C10—C1—H1	107.3033	C16—C15—H15	119.2563
C1—C2—C3	120.8 (2)	O5—C16—C15	125.6 (2)
C1—C2—C7	120.2 (3)	O5—C16—C17	114.9 (2)
C3—C2—C7	119.0 (3)	C15—C16—C17	119.5 (3)
C2—C3—C4	121.5 (3)	O6—C17—C16	115.6 (2)
C2—C3—H3	119.2635	O6—C17—C18	125.5 (3)
C4—C3—H3	119.2645	C16—C17—C18	118.9 (3)
O2—C4—C3	125.8 (3)	C17—C18—C19	121.8 (3)
O2—C4—C5	115.0 (2)	C17—C18—H18	119.0863
C3—C4—C5	119.2 (3)	C19—C18—H18	119.0868
O3—C5—C4	115.5 (2)	C14—C19—C18	118.8 (3)
O3—C5—C6	125.4 (2)	C14—C19—C20	121.1 (3)
C4—C5—C6	119.0 (3)	C18—C19—C20	120.0 (2)
C5—C6—C7	121.5 (3)	C19—C20—C21	111.2 (2)
C5—C6—H6	119.2431	C19—C20—H20a	109.4705
C7—C6—H6	119.242	C19—C20—H20b	109.4713
C2—C7—C6	119.7 (3)	C21—C20—H20a	109.4716
C2—C7—C8	121.3 (3)	C21—C20—H20b	109.4716
C6—C7—C8	119.0 (2)	H20a—C20—H20b	107.6461
C7—C8—C9	112.8 (2)	N2—C21—C20	108.8 (2)
C7—C8—H8a	109.4715	N2—C21—H21a	109.4714
C7—C8—H8b	109.4718	N2—C21—H21b	109.471
C9—C8—H8a	109.4708	C20—C21—H21a	109.4707
C9—C8—H8b	109.4709	C20—C21—H21b	109.4716
H8a—C8—H8b	105.924	H21a—C21—H21b	110.1482
N1—C9—C8	108.1 (2)	O4—C22—C13	107.5 (2)
N1—C9—H9a	109.4715	O4—C22—H22a	109.4708
N1—C9—H9b	109.4715	O4—C22—H22b	109.4708
C8—C9—H9a	109.4708	C13—C22—H22a	109.4715
C8—C9—H9b	109.4708	C13—C22—H22b	109.4715
H9a—C9—H9b	110.8097	H22a—C22—H22b	111.3954
O1—C10—C1	110.4 (2)	O5—C23—H23a	109.471
O1—C10—H10a	109.4717	O5—C23—H23b	109.4715
O1—C10—H10b	109.4714	O5—C23—H23c	109.4711
C1—C10—H10a	109.4713	H23a—C23—H23b	109.4715
C1—C10—H10b	109.4709	H23a—C23—H23c	109.4704
H10a—C10—H10b	108.4859	H23b—C23—H23c	109.4719
O2—C11—H11a	109.471	O6—C24—H24a	109.4707
O2—C11—H11b	109.4716	O6—C24—H24b	109.4717
O2—C11—H11c	109.4709	O6—C24—H24c	109.4719
H11a—C11—H11b	109.4714	H24a—C24—H24b	109.4711
H11a—C11—H11c	109.4708	H24a—C24—H24c	109.4706
H11b—C11—H11c	109.4716	H24b—C24—H24c	109.4714

### Hydrogen-bond geometry (Å, °)

**Table:**

*D*—H···*A*	*D*—H	H···*A*	*D*···*A*	*D*—H···*A*
O1—H1o···N2^i^	0.83 (3)	1.92 (3)	2.740 (4)	169 (3)
O4—H4o···N1^ii^	0.79 (3)	2.03 (3)	2.810 (4)	172 (4)

Symmetry codes: (i) −*x*, *y*+1/2, −*z*+1/2; (ii) −*x*+1, *y*−1/2, −*z*+1/2.
